# An efficient sorghum protoplast assay for transient gene expression and gene editing by CRISPR/Cas9

**DOI:** 10.7717/peerj.10077

**Published:** 2020-10-13

**Authors:** Ruirui Meng, Chenchen Wang, Lihua Wang, Yanlong Liu, Qiuwen Zhan, Jiacheng Zheng, Jieqin Li

**Affiliations:** College of Agriculture, Anhui Science and Technology University, Fengyang, China

**Keywords:** Sorghum, Protoplast, Transient gene expression, Gene editing, CRISPR/Cas9

## Abstract

Protoplasts are commonly used in genetic and breeding research. In this study, the isolation of sorghum protoplasts was optimized and applied to transient gene expression and editing by CRISPR/Cas9. The protoplast was most viable in 0.5 M mannitol, which was the highest of three concentrations after 48- and 72-hours treatments. Using this method we can derive an average of 1.6×10^6^ cells which vary from 5 to 22 nm in size. The average transfection of the protoplasts was 68.5% using the PEG-mediated method. The subcellular assays located Sobic.002G279100-GFP and GFP proteins in the cell compartments as predicted bioinformatically. Two CRISPR/Cas9 plasmids were transfected into sorghum protoplasts to screen for an appropriate sgRNA for gene editing. One plasmid can correctly edit the target region using a single protoplast cell as template DNA. Our results indicated that the protoplast assays as optimized are suitable for transient gene expression and sgRNA screening in CRISPR/Cas9 gene editing procedures.

## Introduction

A protoplast is a plant cell after the cell wall is removed. The wall is typically removed enzymatically or mechanically. Protoplasts provide a unique single cell system for genetics researches. Recently, plant protoplast system has been adapted in a series of studies for gene function (Yanagisawa & Sheen, 1998), nucleo-cytoplasmic interactions (Yu et al., 2014), and gene responses to stress (Chen et al., 2015).

Sorghum (*sorghum bicolor*) is a multi-functional crop and an ideal model for C4 crops because of its small genome (Paterson et al., 2009). There are a few reports about sorghum protoplast isolation focusing mainly on the culturing and regeneration of plants from protoplasts. For example, Yu et al. (2014) used sorghum protoplasts in plant regeneration. Compared with rice and maize, there are fewer reports about gene function research using sorghum protoplasts.

The CRISPR (Clustered Regularly Interspaced Short Palindromic Repeats)/Cas system is a newly developed genome editing technology (Cong et al., 2013). It uses defense systems found in prokaryotic organisms to fight against foreign nucleic acids. A single nuclease Cas9 and a single guide RNA (sgRNA) can be combined as an efficient gene editing system to cleave cognate DNA homologous to the spacer (Cong et al., 2013). This system has been most commonly used in editing the genomes of many crops including rice, maize, barley and sorghum (Miao et al., 2013; Li et al., 2017; Jiang et al., 2013; Kapusi et al., 2017). It has become a powerful tool in plant genetics research. One example is chlorophyll *a* oxidase (CAO) which catalyzes the conversion of chlorophyll *a* into chlorophyll *b*, one of the rate-limiting enzymes in chlorophyll synthesis (Reinbothe et al., 2006). Knocking this gene out turn the leaves yellow. So, it was used as a common target gene for CRISPR/Cas9 in rice (Begemann et al., 2017).

In this study, we developed a highly efficient sorghum protoplast isolation assay from young green tissue and applied the isolated protoplasts to the transient gene expression and gene editing by CRISPR/Cas9 in sorghum.

## Materials and Methods

### Protoplast isolation

Seeds of sorghum cultivar Tx623 were planted in pots and then incubated with a photoperiod of 12 h light and 12 h dark at 28 °C for 10–15 days. Green tissue from stem were cut in approximately 0.5  mM strips using a razor blade. The strips were incubated in D solution (10 mM KCl, 8 mM MES,1 mM CaCl_2,_pH 5.7) with different mannitol concentrations. The enzyme solution contained D solution with 0.6% cellulose, 0.1% pectolyase, 0.1% BSA, and 0.1% polyvinylpyrrolidone K30. one g of the strips were added to 10 mL of the enzyme solution, which was incubated for 4 h in the dark at room temperature and agitated at 40 rpm. An equal volume of W5 (154 mM NaCl, 125 mM CaCl_2_, 5 mM KCl, and 2 mM MES, pH 5.7) solution was added and the mixed solution was shaken for 1 h at 80 rpm. The mixed solution containing protoplasts was then filtered through a 75 nm nylon mesh into a 50 ml tube and centrifuged at 1200 rpm for 5 min to collect the protoplasts. The enzyme solution and W5 solution were sterilized by a 0.22 µm filter before use.

### Plasmid construction

The pAN580 plasmid was used to perform subcellular localization. The gene *Sobic.002G279100* with XbaI and BamHI sites were cloned from sorghum cDNA without a stop codon and were inserted into pAN580. The primer sequences are shown in Table S1. Sobic.002G279100 was predicted to be localized in the nucleus using Target P 2.0 server (http://www.cbs.dtu.dk/services/TargetP/), with the Organism group parameter set to plant.

**Figure 1:**
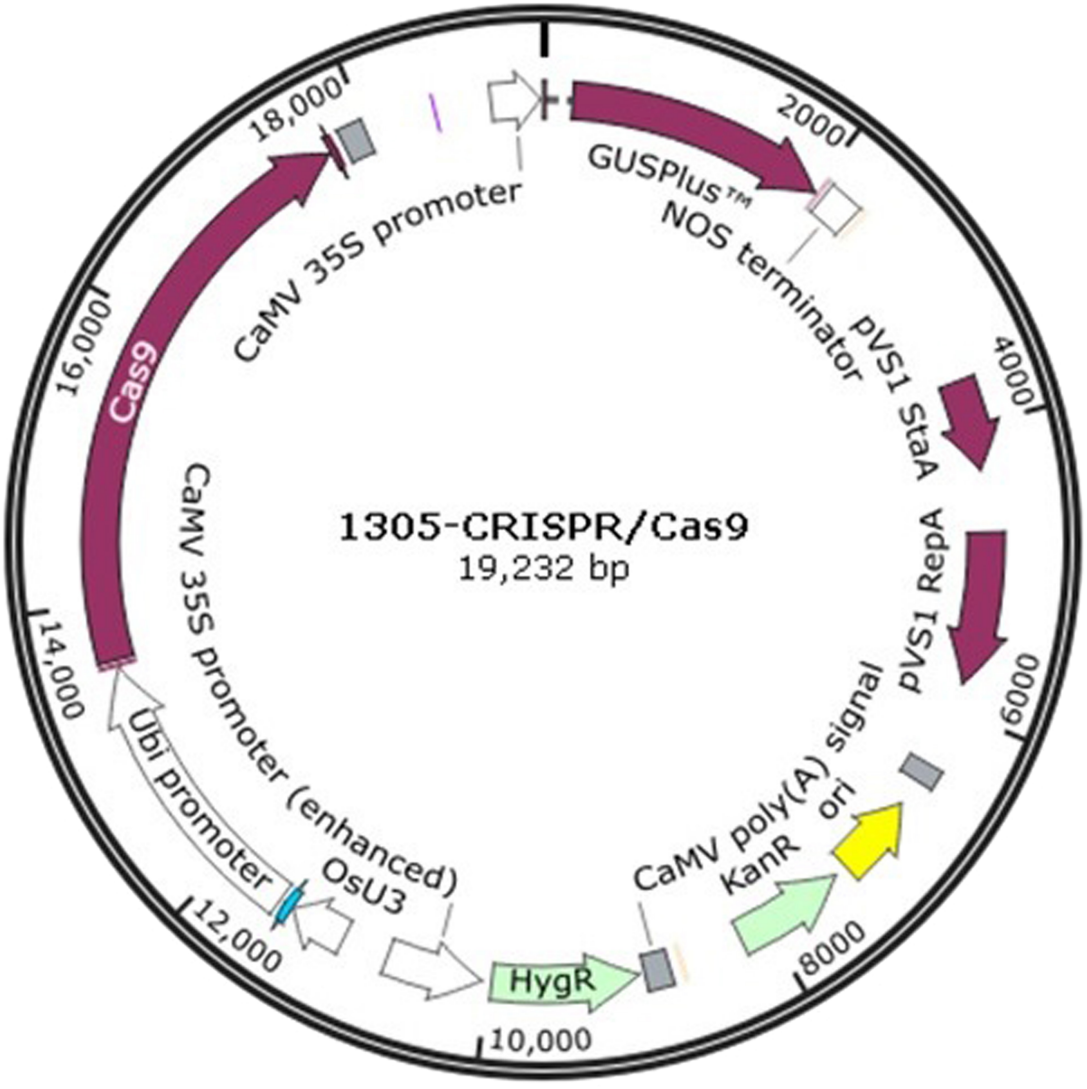
The plasmid map of 1305-CRISPR/Cas9.

The 1305-CRISPR/Cas9 plasmid (Fig. 1) was used to perform sorghum gene editing. The target gene chlorophyllide *a* oxygenase (CAO) was selected. The two sgRNAs primers, CAO1 and CAO2, were designed as shown in supplementary Table 1. The primers, CAO1-f and CAO1-r, were mixed together and diluted to 10 mM concentration. The solution was heated at 95 °C for 5 min and cooled at room temperature. The plasmid was digested with *Ara* I and was ligated with the heated primers to generate 1305-CRISPR/Cas9-CAO1. The same strategy was used to generate 1305-CRISPR/Cas9-CAO2.

**Table 1:** The effects of different mannitol concentrations.

Manitol Concentration(M)	The percentage of protoplast alive in 48 h	The percentage of protoplast alive in 72 h	t
0.4	39.7% ± 4.5% c	18.3% ± 3.5% c	6.46^*^
0.5	89.0% ± 3.6% a	86.0% ± 5.3% a	0.81
0.6	55.7% ± 5.1% b	50.1% ± 2.2% b	1.78

**Notes.**

The different lower letters represent the difference at 0.05 level.

* represents the difference at 0.05 level.

### Protoplast transfection

PEG-transfection was carried out as described in the previous report (Yoo, Cho & Sheen, 2007) with minor revisions. The isolated protoplasts were suspended in 600 µL suspension solution (0.4M mannitol, 20 mM CaCl_2_, 5 mM MES, pH 5.7). Ten µg plasmid DNA was added to 200 µL protoplast suspension in a two mL microcentrifuge tube. Two hundred twenty µL 40% PEG solution (40% PEG 4000, 0.1M CaCl_2_, 0.4M mannitol, pH 5.7) was added and mixed immediately by gentle shaking. The mixed solution was incubated for 20 min at 28 °C. Then 440 µL W5 was added to dilute the PEG. The protoplasts were collected by centrifugation at 110 ×g for 3 min and suspended in one mL of incubation buffer (0.5M mannitol, 4 mM KCl, 4 mM MES, pH 5.7). The incubation solution was stored at 28 °C for 48–72 h.

### Confocal laser scanning microscopy

Protoplasts were observed using a confocal laser scanning microscope (Leica, Germany) after transfection for 48–72 h. GFP and chloroplasts were excited at 488 nm and 514 nm. The emission filters of GFP and chloroplasts were 500–530 nm and 650–750 nm, respectively.

### Mutation validation in protoplasts

The protoplasts were transfected after three days. The number of protoplasts was measured by a hemocytometer. After transfection, the protoplasts were diluted to 1 protoplast per µL. Then 1 µL protoplast solution was transferred to the PCR tube and checked for protoplast content using a microscope. Tubes with a protoplast cell underwent PCR using KOD FX polymerase (Toyobo, Osaka, Japan). To obtain sufficient product, two rounds of PCR were performed. The amplification primers CAO1-1 were as shown in supplementary Table 1. The first PCR round was as follows: one cycle of 94 °C for 5 min, followed by 36 cycles of 30 s at 94 °C, 30 s at 55 °C and 60 s at 68 °C, and a final extension at 68 °C for 7 min. The products from the first round provided the template DNA for the second round PCR using the same thermocycling. The PCR products from the second round were digested by T7 endonuclease I (NEB, UK) at 37° for 30 min. The digested products were detected by 1% agar gel and sequenced by Genscript Company (Nanjing, China).

### Data analysis

The three replicates data for the percentage of protoplast alive was used for statical analysis by Excel 2010. Two tailed t test was conducted. The null hypothesis was that the mean percentage of protoplast alived in 48 and 72 h was equal.

## Results

## Optimization of protoplast isolation

The osmolality is a key factor to maintain protoplast viability after enzyme hydrolysis (Lei et al., 2015), therefore the concentration of mannitol should be optimized. The results showed that protoplast viability in 0.5 M mannitol is the highest in the three concentrations after 48 and 72 h treatments (Table 1). The viability showed no significant difference between the 48 h and 72 h treatment in a 0.5 M mannitol concentration. Therefore, the optimal mannitol concentration was 0.5 M in the enzyme solution.

**Figure 2:**
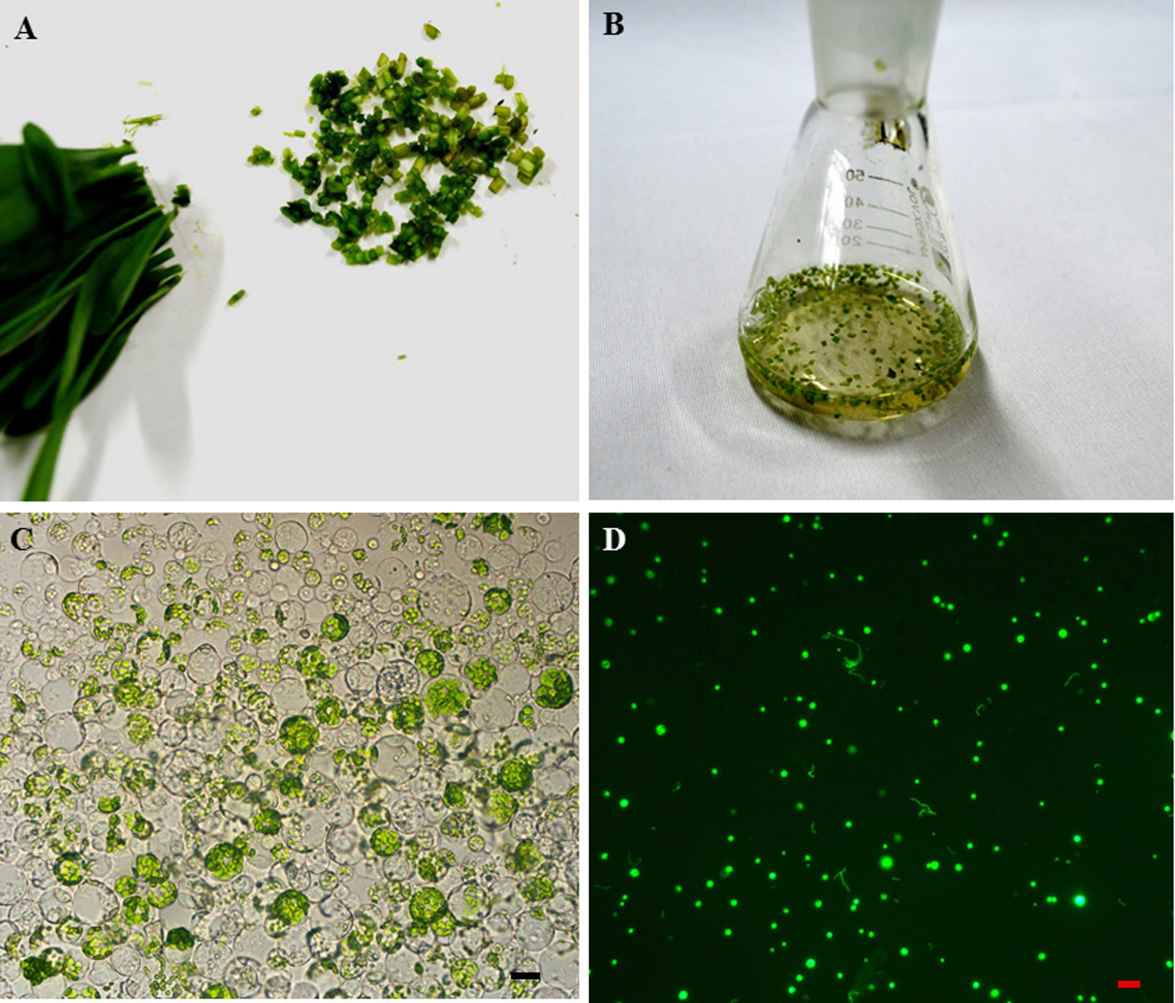
The sorghum protoplast isolation and transformation. (A) The seedlings were cut into 0.5 mm strips. (B). Strips treated with enzyme solution. (C) The image of protoplasts was obtained under a microscope, scale bar = 10 µm. (D) The transfection image of protoplasts was obtained under a fluorescent microscope.

To establish an efficient protoplast isolation assay, 10 to 15 day old seedlings were used to isolate the protoplast. The stems were selected and cut into about 0.5 mm strips (Fig. 2A). The fresh strips were immediately transferred into an enzyme solution with a 0.5 M mannitol concentration (Fig. 2B). This treatment can derive an average of 1.6 ×10^6^ cells which vary from 5 to 22 nm in size (Fig. 2C). The GFP fluorescence was clearly detected in the protoplast after transfection with the 35S::GFP plasmid by the PEG-mediated transfection method and an incubation period of 24 h (Fig. 2D). There were 120, 136 and 155 protoplasts with GFP fluoresence in two hundred protoplasts, individually. The average transfection was 68.5% in the sorghum protoplast (Fig. 2D).

### Subcellular localization studies in sorghum protoplast

**Figure 3:**
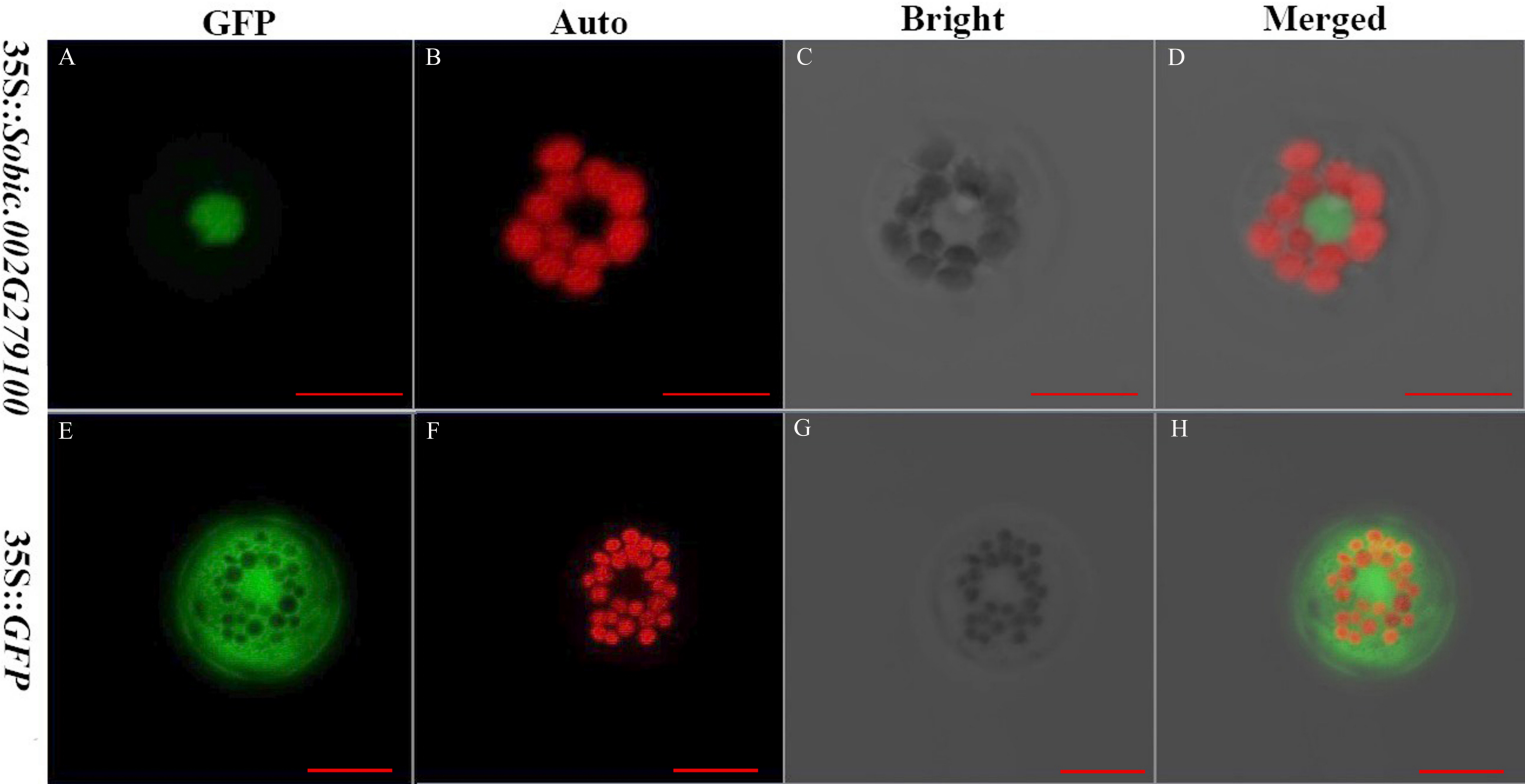
The subcellular localization of two genes using sorghum protoplasts (scale bar = 10 µm). (A) Fluorescence of Sobic.002G279100-GFP. (B) Chlorophyll auto-fluorescence. (C) A protoplast in bright. (D) Merged images of Sobic.002G279100-GFP and Auto ones in bright. (E) Fluorescence of GFP. (F) Chlorophyll auto-fluorescence. (G) A protoplast in bright. (H) Merged images of GFP and Auto ones in bright.

The subcellular localization of proteins is crucial because it provides the physiological context for gene function (Hung & Link, 2011). The sorghum gene *Sobic.002G279100* fused with GFP and GFP alone were expressed in the sorghum protoplasts. Sobic.002G279100 was predicted to be localized in the nucleus using Target P server. GFP was a protein localized in the cytoplasm. The results showed that Sobic.002G279100-GFP was localized in the nucleus as predicted (Fig. 3). Our results indicated that the sorghum protoplast system is suitable for subcellular localization.

### CRISPR/Cas9 editing of sorghum protoplasts

**Figure 4:**
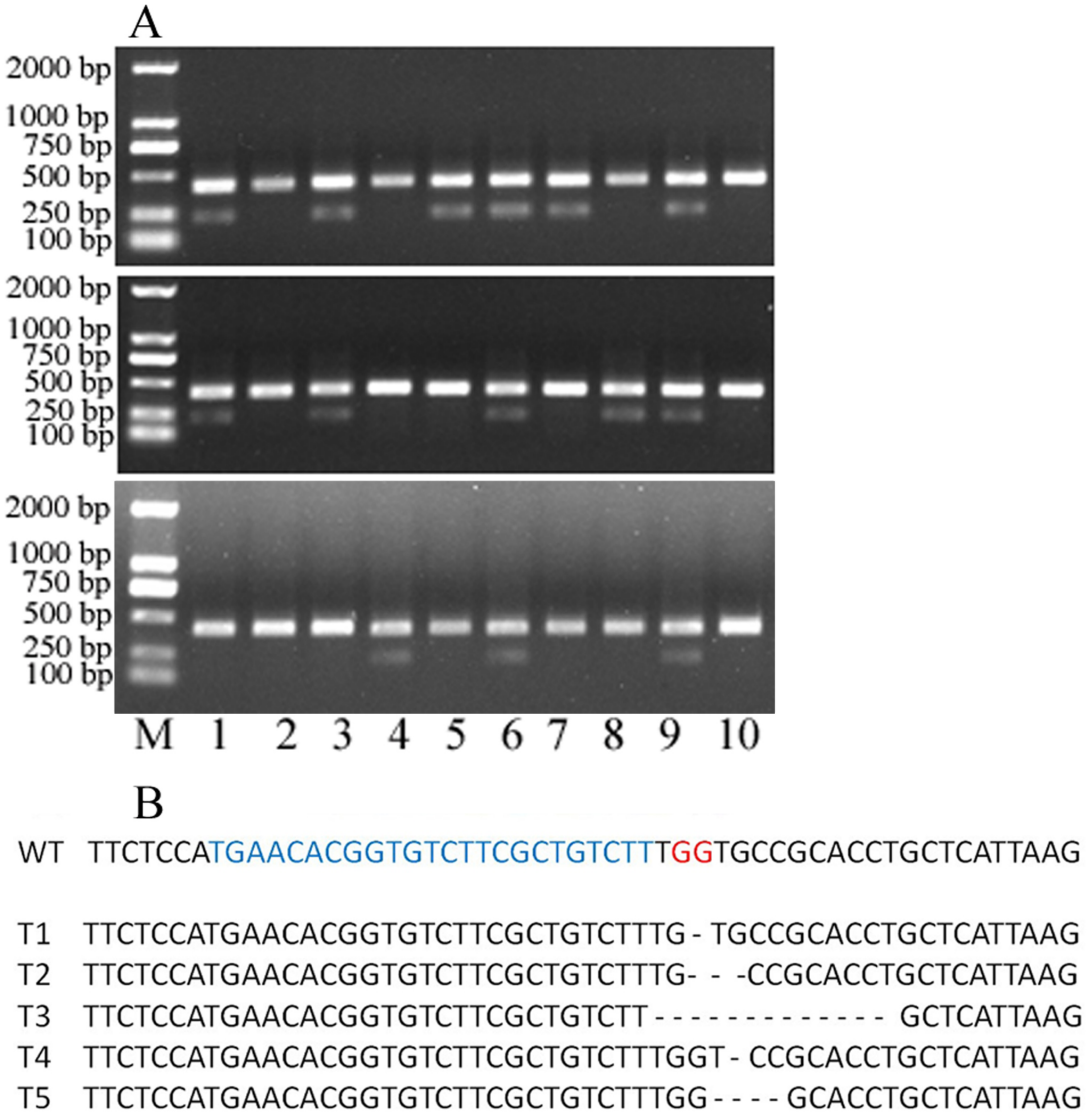
30 single protoplasts with T7E1 mismatch detection assay and sequencing results. (A) T7E1 mismatch detection assay for 30 single protoplasts; M represents marker. (B) The sequencing results of editing protoplast and wild type; WT, wild type; blue letters represent sgRNA; red letters represent PAM; - represents deleted nucleotides.

Sorghum is considered to be a recalcitrant major crop in terms of tissue culture and genetic transformation (Liu & Godwin, 2012). An appropriate sgRNA is a key factor to knock out genes using the CRISPR/Cas9 system (Zhu et al., 2016). Therefore, it is important to screen for an appropriate sgRNA for the further genetic transformation of sorghum. The two plasmids 1305-CRISPR/Cas9-CAO1 and 1305-CRISPR/Cas9-CAO2 were separately transfected into the sorghum protoplasts. Only 1305-CRISPR/Cas9-CAO1 knocked out the target region (Fig. 4B).

To evaluate the knocking-out efficiency, 30 single protoplast cells were separately transferred into tubes and detected by T7 endonuclease 1(T7E1) mismatch detection assay. It showed that 14 protoplasts can be digested by T7 endonuclease 1 (Fig. 4A). These protoplasts cells showed five types of editing in the target gene region (Fig. 4B, Fig. S1). This indicated that the editing efficacy was 46.7%. Above all, the protoplast assay can be a suitable system to screen sgRNAs and evaluate their editing efficiency.

## Discussion

Protoplasts have been widely used in genetic transfection and transient gene expression. Previously, there were a few reports about sorghum protoplast isolation (Sairam et al., 1999; Karunaratne & Scott, 1981). We optimized the sorghum protoplast isolation assay, which was based on the protoplast isolation assay of maize (Zelazny et al., 2007). Our results indicated that the system has a high protoplast yield and an efficient transfection rate and therefore is suitable for transient gene expression and gene editing using CRISPR/Cas9.

CRISPR/Cas9 has become a powerful genome editing tool for plant breeding and genetic research because of its simplicity and versatility (Cong et al., 2013). The editing efficiency still needs to be improved because it is affected by the Cas9 version and sgRNA (Murovec, Pirc & Yan, 2017; Shimatani et al., 2017). An appropriate sgRNA is a key factor to editing efficiency, but sgRNA targeting efficiency still relies on empirical results. Therefore, a rapid and efficient method to identify sgRNA becomes very important, especially for a recalcitrant crop such as sorghum. In this study, we applied CRISPR/Cas9 to edit the CAO gene in sorghum protoplasts. The process of protoplast transformation and editing verification takes approximately 3 to 5 days. Comparing to transgene, it is an easy way to identify the efficiency for sgRNA.

Many studies had been done using protoplasts to evaluate the efficiency for different CRISPR systems. After the CRISPR/Cas9 plamsmid was transferred into protoplasts, total DNA were extracted from transfected protoplasts. Then mutagenesis frequency from the target was evaluated by Restriction Fragment Length Polymorphism (RFLP) (Feng et al., 2013; Nekrasov et al., 2013; Shan et al., 2013) or the T7 endonuclease 1(T7E1) mismatch detection assay (Kim et al., 2017; Woo et al., 2015). Lin et al. (2018) showed that single-cell (protoplast) analysis is a sensitive and convenient method to evaluate the efficiency of various sgRNAs. In this study, we also applied the single-cell analysis to identify the editing target and to evaluate the editing efficiency. Usually, it will take 77-112 days from initating immature embryos to planting putative transgenic plantlets (Liu & Godwin, 2012). Wood et al. (2009) has adapted a transient gene expression system in Nicotiana bethaminan leaf to simultaneously express five genes and test their phenotypes in five days. Compared to these platforms, our system only takes three days to identify the editing region. So, our results demonstrated that this is a rapid and efficient evaluation method for screening sgRNAs in sorghum.

Our system also had limitations in application for the subcelluar localization of proteins and screening sgRNAs. Transient gene expression is commonly used in gene function research using target proteins fused to fluorescent tags (Zhang et al., 2011). In this study, both tagged and untagged proteins were targeted at the predicted compartments in the sorghum cells. Therefore, the system can be used in the subcellular localization of proteins although the assay cannot be applied to the subcellular localization of proteins which are targeted at the cell wall because protoplasts lack cell walls. In the research, we applied two rounds of PCR to amplify enough DNA to sequence as the amounts of DNA in a single cell were very low. In heterozygous gene editing condition, increased in PCR cycles may result in biased amplification of one allele over the other. So, it is necessary to identify the editing region by both T7 endonuclease 1 mismatch detection assay and sequencing.

## Conclusions

In this research, we optimized the isolation of sorghum protoplast and applied to transient gene expression and editing by CRISPR/Cas9. The best mannitol concentration is 0.5 M for the isolation of sorghum protoplasts. The subcelluar assys showed that GFP and Sobic.002G279100-GFP were individually located in the cell compartments as we predicted by bioinformatic software. We also screened two sgRNAs for CAO gene editing. 14 protplast cells showed five types of editing in the target gene region. It means that the protoplast assay can be suitable system to screen sgRNAs. So, we developed a highly efficient sorghum protoplast isolation assay and applied the isolated protoplasts to the transient gene expression and sgRNA screening.

##  Supplemental Information

10.7717/peerj.10077/supp-1 Supplemental Information 1 The primers used in the research

10.7717/peerj.10077/supp-2 Supplemental Information 2 The sequencing results for the editing target

10.7717/peerj.10077/supp-3 Supplemental Information 3 Table 1 raw data

## Additional Information and Declarations

### Data Availability

The following information was supplied regarding data availability: The raw data used in this research are available in the Supplemental Files.
