# Supplementary material for: An efficient sorghum protoplast assay for transient gene expression and gene editing by CRISPR/Cas9
Source: PeerJ. 2020 Oct 13;8:e10077. doi: 10.7717/peerj.10077 (PMC7566750; doi:10.7717/peerj.10077)
Supplement: Supplemental Information 1 [file peerj-08-10077-s001.docx]

Supplementary table 1 The primers used in the research

| Primer name | Forward primer | Reverse primer |
| --- | --- | --- |
| Sobic.002G279100 | TGCTCTAGAATGGGGAGGTCGCCG3 | CGCGGATCCTTTCATCTCGAGGCT3 |
| CAO1 | GGCATGAACACGGTGTCTTCGCTGTCTT | AAACAAGACAGCGAAGACACCGTGTTCA |
| CAO2 | GGCAGTTCGCCAAGAACAACAACC | AAACGGTTGTTGTTCTTGGCGAAC |
| CAO1-1 | CTCTTATCGACAAGCCCACT | TTTGACGCGAACGAATGA |
