# Supplementary material for: An efficient sorghum protoplast assay for transient gene expression and gene editing by CRISPR/Cas9
Source: PeerJ. 2020 Oct 13;8:e10077. doi: 10.7717/peerj.10077 (PMC7566750; doi:10.7717/peerj.10077)

Supplementary Figure 1: The sequencing results for the editing target.

CK　　　TCTTCGCTGTCTTTGGTGCCGCACCTGCTCA


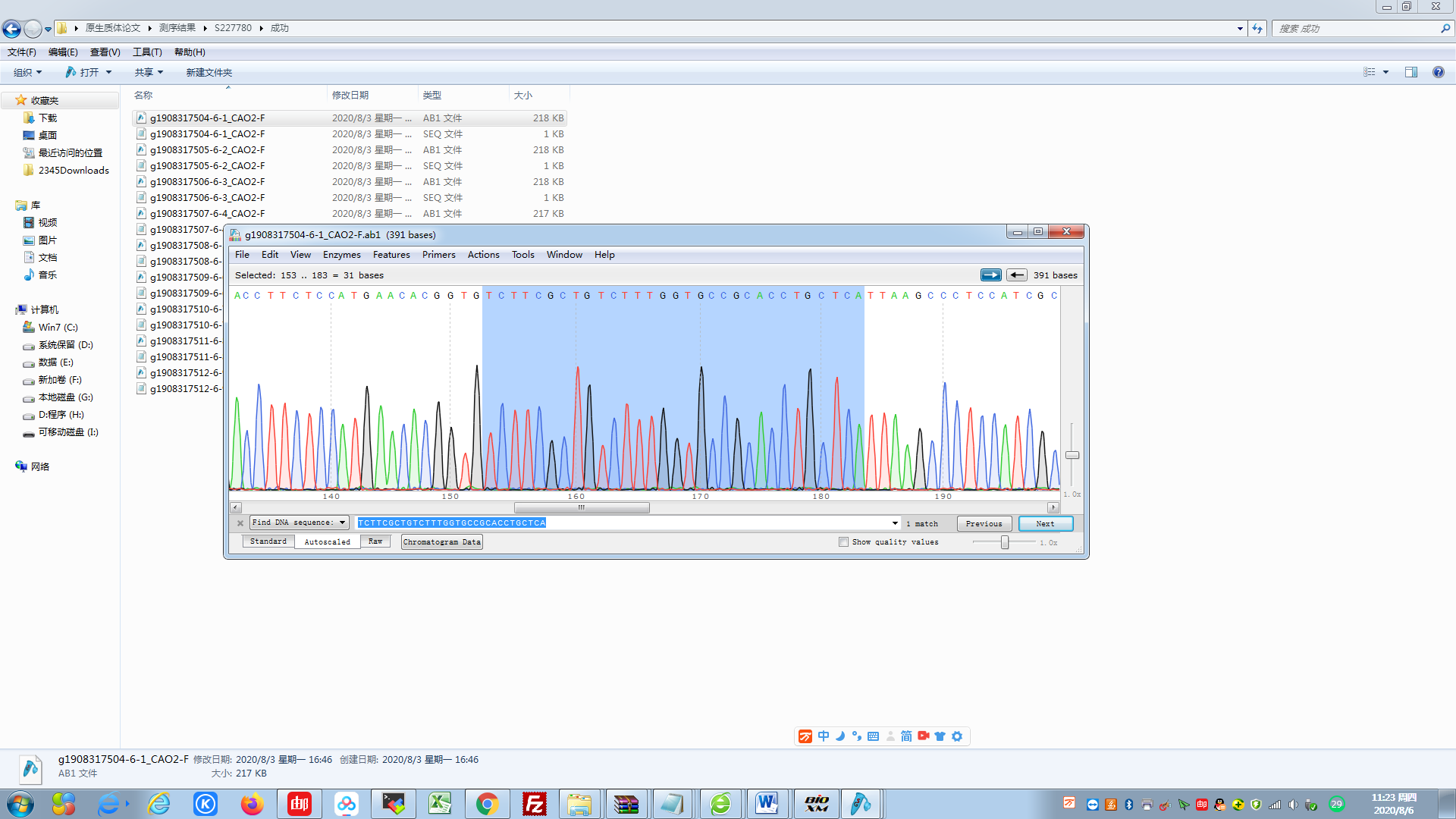


T1 TCTTCGCTGTCTTTGTGCCGCACCTGCTCA (3 protoplasts)


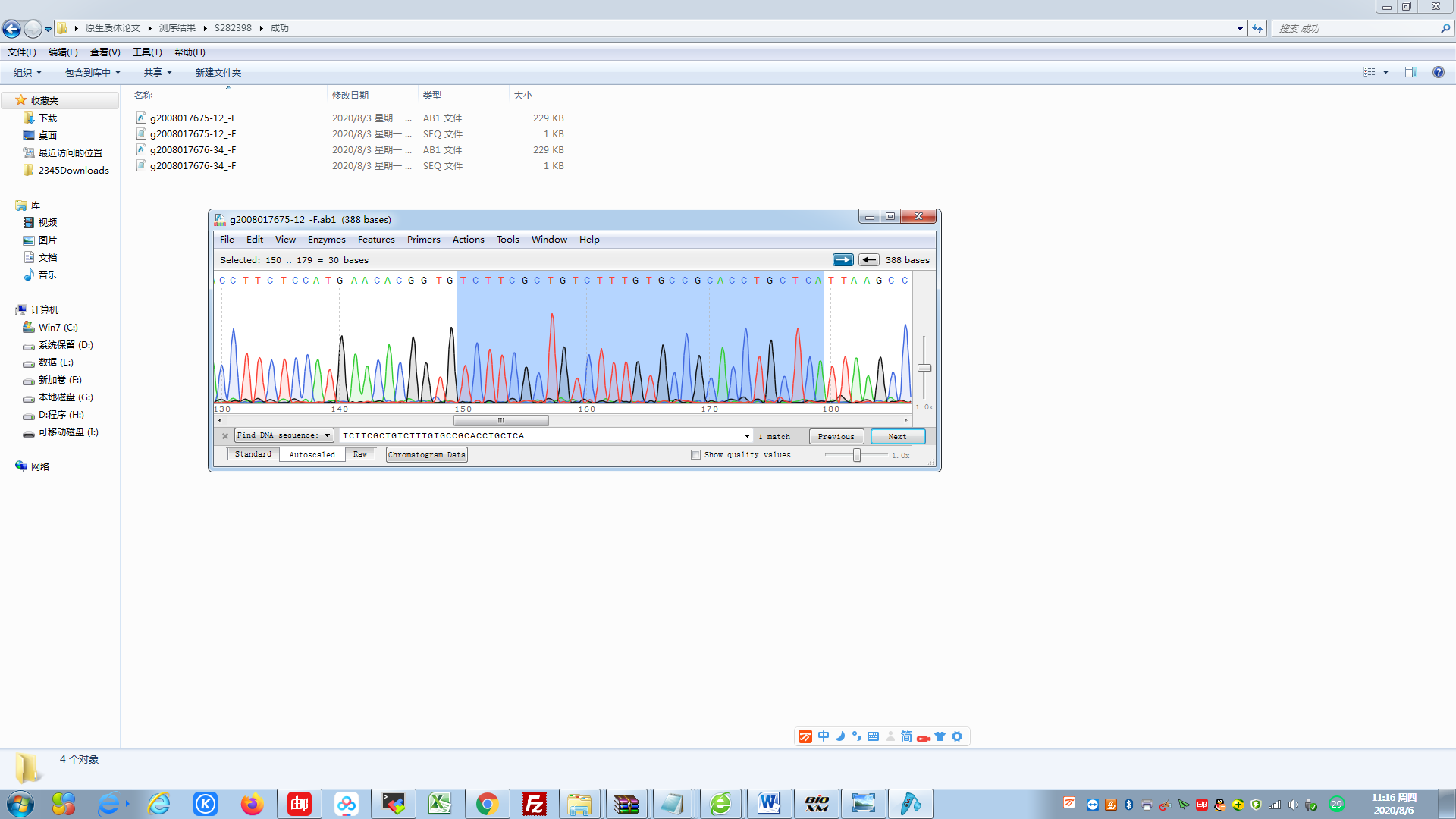


T2 TCTTCGCTGTCTTTGCCGCACCTGCTCA (2 protoplasts)


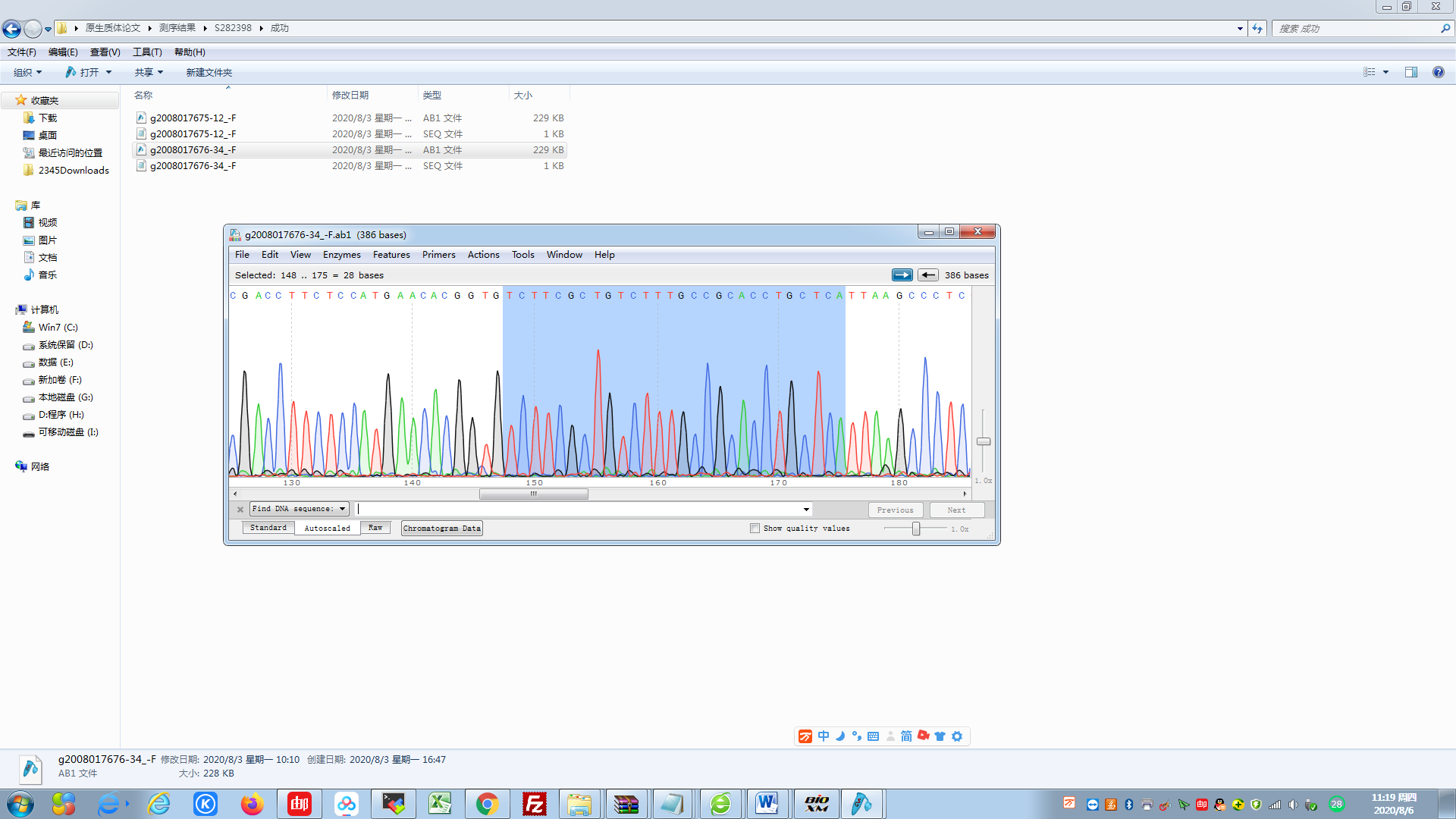


T3 TCTTCGCTGTCTTGCTCATTAAGCCCTC (2 protoplasts)


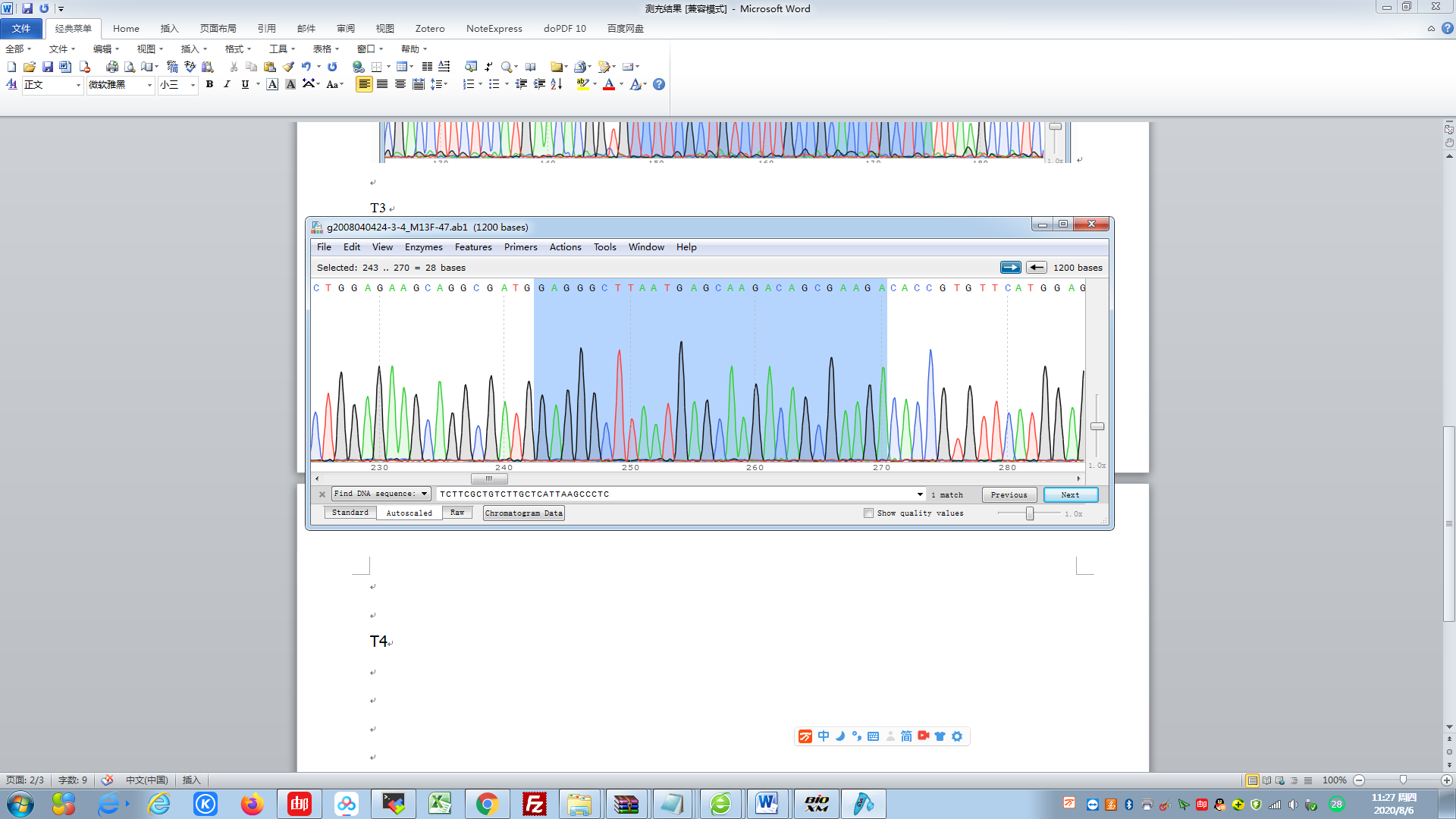


T4　 TCTTCGCTGTCTTTGGTCCGCACCTGCTCA(4 protoplasts)


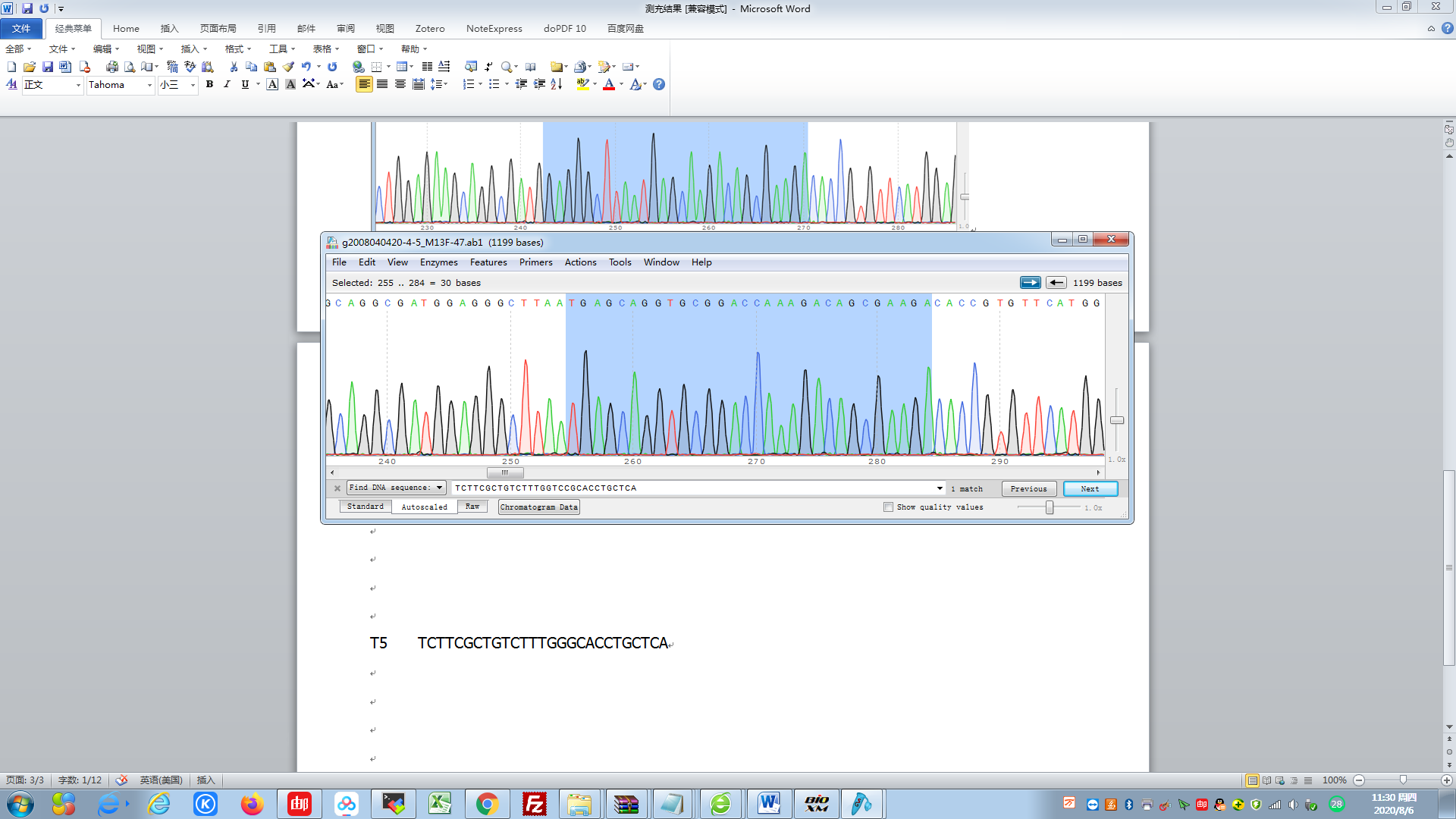


T5　　TCTTCGCTGTCTTTGGGCACCTGCTCA(3 protoplasts)


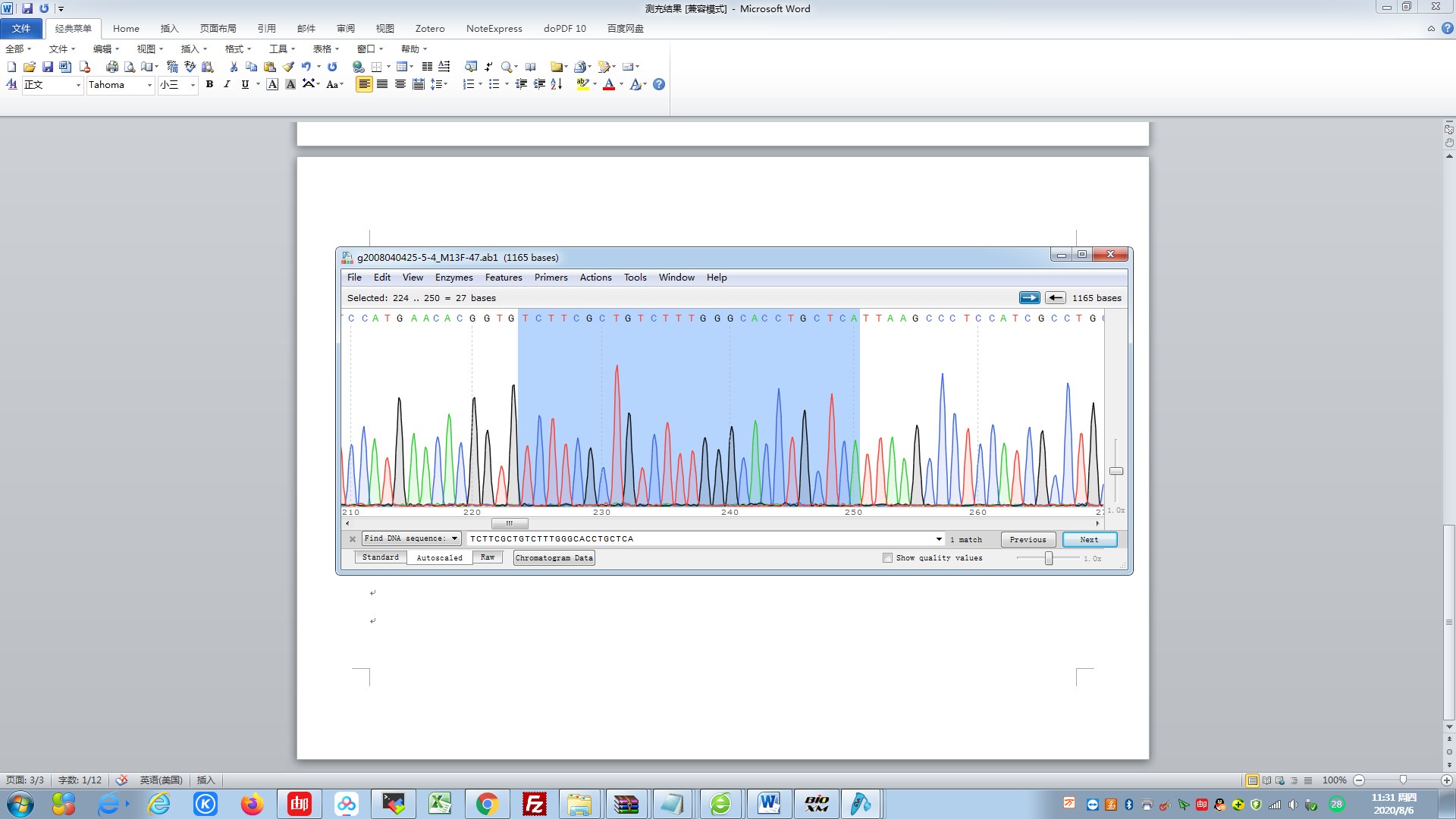

Supplement: Supplemental Information 2 [file peerj-08-10077-s002.docx]
