# Supplementary material for: An efficient sorghum protoplast assay for transient gene expression and gene editing by CRISPR/Cas9
Source: PeerJ. 2020 Oct 13;8:e10077. doi: 10.7717/peerj.10077 (PMC7566750; doi:10.7717/peerj.10077)
Supplement: Supplemental Information 3 [file peerj-08-10077-s003.docx]

Table 1 The raw data of different manitol concentrations

| Mannitol  Concentration | 48 | | | 72h | | | | |
| --- | --- | --- | --- | --- | --- | --- | --- | --- |
|  | Replicate 1 | Replicate 2 | Replicate 3 | Replicate 1 | Replicate 2 | Replicate 3 | |  |
| 0.4M | 0.44 | 0.4 | 0.35 | 0.22 | 0.18 | | 0.15 | |
| 0.5M | 0.9 | 0.85 | 0.92 | 0.9 | 0.8 | | 0.88 | |
| 0.6M | 0.57 | 0.5 | 0.6 | 0.52 | 0.5 | | 0.48 | |
